# The Attitude of Medical and Pharmacy Students towards Research Activities: A Multicenter Approach

**DOI:** 10.3390/pharmacy5040055

**Published:** 2017-10-11

**Authors:** Akshaya Srikanth Bhagavathula, Deepak Kumar Bandari, Yonas Getaye Tefera, Shazia Qasim Jamshed, Asim Ahmed Elnour, Abdulla Shehab

**Affiliations:** 1 Department of Clinical Pharmacy, University of Gondar-College of Medicine and Health Sciences, School of Pharmacy, Gondar 196, Ethiopia; yonas1get@gmail.com; 2 Department of Clinical Pharmacy, Vaagdevi College of Pharmacy, Warangal 506001, Telangana, India; laxmideepak.pharma@gmail.com; 3 Department of Pharmacy Practice, Kulliyyah of Pharmacy, International Islamic University Malaysia, Kuantan 25200, Pahang, Malaysia; shazia_12@yahoo.com; 4 Faculty of Pharmacy, Fathima College of Health Sciences, Al Ain Campus, Al Ain 24162, UAE; asim.ahmed@fchs.ac.ae; 5 Department of Internal Medicine, College of Medicine and Health Sciences, UAE University, Al Ain 17666, UAE; A.shehab@uaeu.ac.ae

**Keywords:** medical students, pharmacy students, attitude, research activities, publications

## Abstract

**Aim:** To assess the attitude of medical and pharmacy students in Asian and African universities towards scholarly research activities. **Methods:** An anonymous, cross-sectional, self-reported online survey questionnaire was administered to medical and pharmacy students studying in various Asian and African universities through social media between May and July 2016. A 68-item close-ended questionnaire consisting of Likert-scale options assessed the students’ research-specific experiences, and their attitudes towards scholarly research publications. **Results:** A total of 512 questionnaires were completed, with a response rate of 92% from Asia and 94% from Africa. More pharmacy students (70.8%) participated than medical students (29.2%). Overall 52.2% of the pharmacy students and 40% of medical students believed that research activities provided a means of gaining respect from their faculty members. Lack of encouragement, paucity of time, gaps in research activities and practices, and lack of research funding were some of the most common barriers acknowledged by the students. A nonparametric Mann-Whitney test showed that a statistically significant difference was observed, in that more than 80% of the pharmacy students viewed scientific writing and research activities as valuable experiences (*p* = 0.001) and would like to involve their co-students in scholarly research activities (*p* = 0.002); whereas the majority of the medical students desired to be involved more in scholarly research publications (*p* = 0.033). **Conclusion:** Pharmacy students had good attitudes towards research activities and a higher number of medical students desired to be involved more in research publications. Faculties may consider taking special research initiatives to address the barriers and improve the involvement of medical and pharmacy students in scholarly research activities.

## 1. Introduction

Providing comprehensive patient care is an important component of all healthcare professions (HCPs). For the provision of effective care, future workers in HCPs are expected to be trained in all aspects, and to exercise proficient skills in their research-based academic education and professional practice. In order to create impact-laden critical reasoning abilities among future healthcare practitioners, research activities should be followed by seminars, conference presentations and publications as part and parcel of every healthcare discipline globally. Thus, one cannot sideline the importance of scholarly research activities as an essential component of a complete medical and health sciences curriculum in undergraduate and postgraduate education. Moreover, the initiation and incorporation of evidence-based knowledge is emphasized globally as an essential component in the modern science education. For the past few decades, a changing trend has been observed regarding the inclusion of research components in medical and pharmacy education [1,2,3,4,5,6,7,8,9,10]. These changes drive the interest among students in conducting research, and presenting and publishing their work at national and international levels. The ability of a student to carry out scholarly research is an added advantage for their academic advancement through acquisition of critical thinking and analytical skills, as well as through comprehension and analysis of the foundations of evidence-based medicine [11,12,13]. Several studies have shown that research experience at a student level is strongly associated with future career achievements and scholarly research initiatives [13,14,15]. Conducting scholarly research activities at student level is an arduous task, and in the context of this, several barriers have been reported, including lack of time, lack of support from faculties, and lack of funding sources, among others [1,11,14,16]. Despite these difficulties and predicaments, medical and pharmacy students perform their research projects across the globe.

Positive attitudes to and opportunities for research activities with adequate provision of facilities and mentorship will equip medical and other health profession students for becoming future healthcare scientists. Early identification of their passion towards research will help to discern their inclination, as well as their potential scope for professional practice in the clinical setting. Most undergraduate medicine and pharmacy programs require coursework in epidemiology, research methodology, biostatistics and literature evaluation [6,7].

However, given the demand and competing interest towards scholarly research, several studies have identified attitudinal ambivalence towards the significance of scholarly research publication [16,17,18,19]. We believe medical and pharmacy students are among the students in the major health profession discipline, who represent potential future leaders in clinical and pharmaceutical research. With this in mind, it is worth studying the attitudes of medical and pharmacy students regarding research activities. Furthermore, a better understanding of medical and pharmacy students’ attitudes, of the barriers involved, and of mentors’ influence, culminating in scholarly research activities and journal publication, is valuable.

The current research is an attempt in this regard, and therefore aims to investigate attitudes towards the scholarly research activities of medical and pharmacy students.

## 2. Methods

This was a cross-sectional survey conducted on medical and pharmacy students enrolled in various Asian (Malaysia, India, Saudi Arabia and the United Arab Emirates) and African universities (Ethiopia, Kenya and Egypt). A web-based survey through anonymous questionnaire was administered during the period May–July 2016. This online survey was designed and primarily used to gather data about students’ scholarly research activities through internet and social networking sites, as well as through personal emails. The questionnaire was focused on medical and pharmacy students via an online survey instrument tool. Furthermore, e-mails carried a Uniform Resource Locator-URL link to the online survey developed and distributed through social network sites like Facebook, LinkedIn, and Twitter to encourage student participation.

Medical and pharmacy students who were enrolled at Asian and African universities were the source population. The targeted population was senior medical and pharmacy students, who were randomly selected from various universities.

An online sample size calculator—“Creative research systems” [17,19]—was used to determine the number of participants for the survey, by considering 95% confidence level with an accuracy of 50% for a student population size of 135,000 across various universities; given a confidence interval of 4.2, the recommended sample size was 542 or more. Estimating a dropout rate of 10–15%, a total of 620 students were invited to participate in the survey. Participation within these representative samples was completely voluntary, and confidentiality was maintained at all stages by not disclosing any personal information in the survey results.

The study investigators designed the survey, and the items were adopted and/or modified based on a review of the literature [1,2,3,6,7,13,14,15,16,17,19,20]. The survey questionnaire was developed in English, and tested for reliability, psychometrics, internal content and construct validity in a methodological, structured approach. A Cronbach alpha exploratory factor analysis was used as a measure of reliability. The internal consistency estimate of the reliability of the test (Cronbach’s alpha) was found to be 0.76, indicating a good construct. The questionnaire was pretested in fifteen percent of the total sample size, which was not included in the study. Further, any ambiguous and unsuitable questions were modified for the final questionnaire.

The study questionnaire consisted of 68 close-ended questions subdivided into 3 categories. The first part included the socio-demographic characteristics, and contained 10 items, including age, gender, region of origin, type of studentship (medical/pharmacy), academic year, living area, type of institution, previous research grant experience, the time dedicated for research grant searching, and the number of scholarly research publications. Furthermore, the second part was comprised of 4 domains, which included a 3-point Likert scale of their priorities, which highlighted their preferences for the type of research articles that were interested in publishing (8 items), their reasons for practicing research publishing (9 items), and the important obstacles to conducting research (10 items). In addition, 12 items focused on their preferences regarding writing for publications, as well as the types of journals in which they preferred to publish their scholarly research activities (9 items). The third section contained 10 items related to their opinions towards the value of scholarly research publications, and were assessed using a 4-point Likert scale (1-strongly disagree to 4-strongly agree). The survey took an average of ten to fifteen minutes to complete.

A statistical analysis was performed using Statistical Package for Social Sciences (SPSS) version 22 for Windows. Descriptive statistics were used to describe demographics and research background experiences. For ease of reporting, differences using agreement responses of high priority (i.e., responses with “agree” and “strongly agree”) were grouped together. Disagreement responses of low priority, “strongly disagree” and “disagree” of the Likert scale, were utilized. Mann-Whitney (M-W) and Chi-square tests were conducted to further analyze their opinions on possible perceptions towards scholarly research activities. Statistical significance was based on a *p*-value of <0.05.

Ethical approval for conducting the study was obtained from the Institutional review board of the School of Pharmacy, University of Gondar, Ethiopia. Written informed consent was obtained from each participant prior to the administration of the study questionnaire. Confidentiality of the information of the respondents was strictly maintained.

## 3. Results

A total of five hundred and twelve student participants completed the survey questionnaire, with response rates of 92% from Asia and 94% from Africa. A higher percentage of pharmacy students (70.8%) participated in the survey than medical students (29.2%). The mean age of individuals sampled is 23 ± 1.42 years (range = 19–30), with 324 males (63.2%). In particular, the majority of the participants were from the fourth year of pharmacy (54.6%), studying in public universities (65.1%), and living outside their study campus (54.8%). Regarding interest towards scholarly research activities, only 19.1% (98/512) had received a research grant for conducting their research, and 48.6% of the medical and pharmacy students stated that they did not dedicate time to searching for grants. In addition, 72.5% did not publish any scholarly research (Table 1).

Nearly 43% of both medicine and pharmacy students agreed that they were interested in focusing on original research, and 4.7% of both medical and pharmacy students were interested in systematic review studies, with a slightly higher preference by pharmacy students (*p* < 0.004). A significant number of students shared that the reasons for interest in scholarly research publications was to improve their relationships with and gain respect from faculty members (48.6%), to improve writing and research skills (44.1%), and to advance their career opportunities (42%). Nearly forty percent of the respondents from both medical and pharmacy groups felt that lack of support from their faculties, lack of time for conducting research and existence of gaps within research activities to their practice were some of the perceived barriers to conducting research. (Table 2).

The results of the Chi-square test for difference between medical and pharmacy students in terms of their opinion on different types of research activity interests showed that there was a significant difference based on student disciplines. Interestingly, significant differences were noticed in relation to the type of research activities, except for data analysis (*p* > 0.05), the publication process (*p* > 0.05) and recognition for their research work (*p* > 0.05) (Table 3). However, the differences were significant for reviewing literature, data interpretation, writing manuscripts, collaborative research with different faculties, research learning experiences, sense of satisfaction for their research work, sharing their research ideas, and students’ experiences for conducting research. Thus, the null hypothesis was accepted for three items, but rejected for these eight items. There was a difference between pharmacy and medical students for ‘data interpretation’ (*p* > 0.001) and ‘experience for conducting research’ (*p* > 0.001). Students were asked to indicate the type of journals preferred for scholarly publication using a 3-point Likert scale. The Chi-square test for differences between medical and pharmacy students’ opinions on journal preferences suggests there was a statistical difference, except in their preference for open-access paid journals, paid journals with impact factors, free journals with impact factors, and Pubmed- and Scopus-indexed journals (Table 3). Therefore, the null hypothesis for journal selection was accepted for five of the nine items, but rejected in the case of open-access free journals, fast-track publishing journals, Embase indexed journals, and preferring only reputed journals for their scholarly research publications.

Table 4 shows the result of Mann-Whitney *U* test for differences between medical and pharmacy students’ opinions towards the value of research publications and mentor influence. Seventy-six percent of the medical and 78% of the pharmacy students felt that publishing as a student would provide them personal fulfillment. A similar percent reported that their mentors encouraged them to conduct research for publications. Interestingly, a higher proportion of pharmacy students perceived their overall writing for research publication as a good experience (*p* < 0.05), contributing to the literature during student life as a valuable experience (*p* < 005), and would likely encourage their co-students to engage in scholarly research publications (*p* < 0.05). Similarly, a high proportion of medical students perceived that they would like to publish more manuscripts for research publications, but no statistical significance was noted in this context.

## 4. Discussion

In the current research, there are a few notable obstacles reported in terms of conducting and/or performing research. Inappropriate funding, lack of support both in kind and cash from the mentors and the institutes, and paucity of time are the hallmarks of non-accomplishment of research tasks. Aside from these, the limited number of research courses taught within medical and pharmacy curricula is one of the barriers claimed by students to conducting research. These findings mirror the conclusion derived from research in Saudi Arabia and Brazil, which showed that similar factors were predominantly cited as obstacles to conducting research [4,14,21]. Another interesting factor—‘lack of same-gender research mentor’—was also outlined in recently published study by Kharraz and colleagues from Saudi Arabia [21]. Several barriers to scholarly research activities have been identified, such as lack of faculty members with appropriate expertise and sufficient time for mentoring, limited resources, and logistical difficulties [13,22].

The students have shown interest towards original article publications, and reasons for which scholarly publication was considered valuable included improving their relationships with and gaining respect from faculty members, advancing their career opportunities, and improving their writing and research skills. Research skills for pharmacy students (under- and postgraduates) are becoming more important, particularly for obtaining a decent job in a competitive market, and in order to attain scholarships for higher postgraduate studies [7,23,24]. Medical students can be potential contributors to scientific research development through participation in different clinical studies and evidence-based clinical training [8,11,25]. It is noted in the current research that slightly less than half of the respondents reported scholarly research publications as a means to improve the relationship with and gain respect from faculty members, as well as improving their writing and research skills. The motivation itself comes from research regarding whether it would be implemented as a ‘core module’ in future Asian and African medical and pharmacy schools. This can encompass not only research methodology aspects but can also address students’ motivation for doing literature search on one’s own interests, leading to critical appraisal of published studies. This literature review can serve as the backbone of one’s research proposal, which itself contains minute details on executing the research and analyzing the research findings. Moreover, this concept is mirrored in a United States study in which a research elective course on dietary supplements was introduced for pharmacy students. The study findings reported enriched critical reasoning abilities and drug-literature evaluation skills among the student participants, which later contributed towards their practice readiness [24].

The medical and pharmacy schools in many developing countries are not yet offering optimum scholarly scientific writing and research opportunities [19,22,24]. To make it practically possible in Asian and African medical and pharmacy schools, sincere dedication from the faculty in terms of intellectual support and unlimited time can contribute as silently lingering motivators, culminating in the quality of the research, further motivating the student to make it publishable. Interestingly, studies from Germany and Saudi Arabia have reported that the majority of students are motivated to conduct research in order to attain and/or secure research publications [14,25]. In Saudi Arabia, one of the motivators worth mentioning is the mandatory nature of research in curricula [14]. Likewise, for nearly two decades in Germany, medical students have been required to complete a research project followed by a research thesis in order to obtain their medical degree [25]. Nykamp and associates reported that participation of pharmacy students in collaborative scholarly research opportunities with faculty members led to encouraging feedback, personal contentment, and career advancement prospects [13]. These examples can serve as models for Asian and African medical and pharmacy schools for the instituting of research modules as a core aspect in their curricula. The ‘Norwegian Medical Student Research Program’ is a grant scheme for all prospective and aspiring medical students to support doing research in parallel with their studies [26]. This program fortified their research environment, and helped to develop new areas of research by augmenting recruitment towards research. Nevertheless, it also instilled the inspiration to include research in the training of medical doctors [26].

The majority of the medical and pharmacy students in this study felt that publishing as a student would provide them personal fulfillment and a formative training experience, as well as regarding writing for publication to be a good experience; others claimed their mentors encouraged them to conduct research for publications. Cultivating and motivating student participation in research activities in addition to their curricular coursework should be encouraged.

Research sensitization in all undergraduate and graduate students should be advocated to enhance student participation in scholarly research activities, and to equip them with practical research experience to nurture them as future scientists.

We recommend that mindfulness towards research be addressed nationally in every Asian and African country, which could also galvanize research funding resources by means of a clear-cut expected outcome of scholarly publications and presentations in repuyearbook journals and conferences.

## 5. Limitations

A high response rate (>90%) is one of the strengths of the current research, and therefore contributes to the validity and usefulness of the research. Previously published studies reported response rates of not more than 75% [2,11,16,21], and we achieved a highly satisfactory response rate (>90%) [27]. Furthermore, due to the anonymous nature of the current research, the chances of bias are reduced. This does not sideline the importance of limitations, which need to be highlighted in the current research. As in any cross-sectional study, this study also adopted self-reported measures, which are generally subject to recall bias and participants’ exaggerated responses; secondly, despite pilot testing of the instrument, it was not subjected to formal standardization. Due to an underpowered sample in our study, this may contribute to both false-positive and false-negative reporting by rejecting true null hypotheses [28]; therefore, caution should be taken while interpreting our findings. Furthermore, it seems that medical students are underreported in the current research, so non-responders bias is therefore a possibility not to be overlooked. We cannot generalize the findings to pharmacy and medical students on other continents, as this study only focused on those in the Asian and African region.

## 6. Conclusions

The present study shows pharmacy students had good attitudes towards research activities, with a higher number of medical students desiring to engage more in research publications. Faculties should implement special research initiatives to address the barriers and improve the involvement of medical and pharmacy students in scholarly research activities. The current research can serve as motivation to further explore undergraduate students’ opinions towards and experiences of scholarly research activities.

## Figures and Tables

**Table 1:** Demographic characteristics of medical and pharmacy students (*N* = 512).

	Medical (*n* = 150)	Pharmacy (*n* = 362)	Total (%)
Age (years)			
<20	6	14	20 (3.9)
20–25	113	307	420 (82.0)
26–30	31	40	71 (13.8)
>30	0	1	1 (0.1)
Gender			
Male	117	207	324 (63.2)
Female	33	155	188 (36.7)
Academic year			
Fourth	80	280	360 (70.3)
Fifth	39	64	103 (20.1)
Sixth	31	18	49 (9.5)
Living area			
Outside University	44	237	281 (54.8)
Within University	106	125	231 (45.1)
Type of institution			
Public	135	198	333 (65.1)
Private	15	164	179 (34.9)
Received research grant			
Yes	26	72	98 (19.1)
No	124	290	414 (80.8)
Time dedicated for grant searching			
No hours	80	169	249 (48.6)
Daily	16	91	107 (20.8)
Weekly	27	61	88 (17.1)
Monthly	27	41	68 (13.2)
Number of scholarly research publications			
No publications	128	244	372 (72.6)
1–2	20	64	84 (16.4)
3–5	2	28	30 (5.8)
6–10	0	13	13 (2.5)
11–15	0	4	4 (0.7)
16–20	0	3	3 (0.5)
>20	0	6	6 (1.1)

**Table 2:** Attitude of medical and pharmacy students towards research interests (*N* = 512).

Variables	Agreement of High Priority		Percentage	*p* Value
	Medical (*n* = 150)	Pharmacy (*n* = 362)		
Research publications interested				
Original research	54	165	42.7	0.432
Multicenter studies	26	67	18.1	0.456
Randomized controlled trial	17	50	13.1	0.233
Meta-analysis	7	28	6.8	0.182
Systematic review	2	22	4.7	0.004
Observational studies	2	21	4.5	0.100
Comparative studies	2	18	3.9	0.854
Retrospective studies	2	12	2.7	0.105
Reasons for publications				
Advance research/share findings	32	180	41.4	<0.001 ^a^
Interesting/good experience	42	116	30.8	0.041
Was encouraged by teachers	27	179	40.2	<0.001 ^a^
Good achievements/goals	21	57	15.2	0.881
Compulsion from peers	34	177	41.2	<0.001 ^a^
Both Job/Career advancement	45	170	41.9	<0.001 ^a^
Improve writing and research skills	57	169	44.1	0.108
Better chances of to get international recognition	38	118	30.4	0.035
To have a relationship with or to gain respect from faculty members	60	189	48.6	0.010
Obstacles for conducting research				
No research course taught at faculty level	35	115	29.2	0.043
Lack of funding	37	127	32	0.006
Time-limitations	56	138	37.8	0.639
Lack of support, encouragement and rewards	70	131	39.2	0.046
The gap between research activities and practice	64	131	38	0.676
Fear of statistics and data collection	36	83	23.2	0.297
Lack of interest	34	56	17.5	<0.001 ^a^
No research unit at faculty	32	88	23.4	0.643
No research in the field I am most interested in	19	68	16.9	0.734
Relationship obstacles-barriers-difficulties	19	62	15.8	0.121

^a^ Mann-Whitney test was used to determine significance, defined as *p* < 0.05.

**Table 3:** Attitude of medical and pharmacy students regarding type of research activity (*N* = 512).

Students Interest		*N*	Chi-Square	df	*p* Value
Writing for publications					
Review/literature search	Medical	150	4.55	1	0.033
	Pharmacy	362			
Analyzing data	Medical	150	1.681	1	0.195
	Pharmacy	362			
Data interpretation	Medical	150	66.62	1	<0.001 ^a^
	Pharmacy	362			
Writing research report, proposal or manuscript	Medical	150	8.79	1	0.003
	Pharmacy	362			
Collaboration with others or with faculty	Medical	150	7.27	1	0.007
	Pharmacy	362			
The learning experience	Medical	150	24.77	1	<0.001 ^a^
	Pharmacy	362			
Sense of accomplishment/satisfaction/achievement	Medical	150	3.84	1	0.050
	Pharmacy	362			
Publication process, submission, approval and published articles appearance online	Medical	150	0.62	1	0.431
	Pharmacy	362			
Recognition	Medical	150	0.01	1	0.913
	Pharmacy	362			
Sharing ideas/adding to literature/advancing research	Medical	150	13.53	1	<0.001 ^a^
	Pharmacy	362			
A unique experience that is available to only a few students	Medical	150	24.49	1	<0.001 ^a^
Type of Journals preferred for publication	Pharmacy	362			
Open access-paid journals	Medical	150	0.20	1	0.654
	Pharmacy	362			
Open access-free journals	Medical	150	8.68	1	0.003
	Pharmacy	362			
Paid journals with impact factors	Medical	150	2.75	1	0.097
	Pharmacy	362			
Free journals with impact factors	Medical	150	1.59	1	0.207
	Pharmacy	362			
Fast-track publishing journals	Medical	150	9.05	1	0.003
	Pharmacy	362			
Pubmed indexed journals	Medical	150	0.82	1	0.365
	Pharmacy	362			
Science direct/Scopus indexed journals	Medical	150	0.47	1	0.493
	Pharmacy	362			
Embase indexed journals	Medical	150	22.08	1	<0.001 ^a^
	Pharmacy	362			
Only reputed journals (Lancet, Nature etc)	Medical	150	5.57	1	0.018
	Pharmacy	362			

df-differential fraction; ^a^ Chi-square was used to determine the significance, defined as *p* < 0.05.

**Table 4:** Medical and pharmacy students’ attitude regarding the value of publishing and mentor influence (*N* = 512).

Statements	Agreement		*p* Value
	Medical Students (%)	Pharmacy Students (%)	
Publishing as a student provided me with personal fulfillment	76.6%	78.4%	0.257
Contribution to the literature as a student is a valuable experience	66.6%	82.8%	<0.001 ^a^
Publishing is an excellent source of recognition for students	72.6%	78.4%	0.022
Publishing as a student provided me with formative training experience	67.3%	79.0%	<0.001 ^a^
My publication will set me apart from my peers	51.3%	56.0%	0.228
I would like to publish more manuscripts	81.3%	72.6%	0.219
I encourage my co-students to publish	72.6%	81.4%	0.002
Overall, writing for publication is a good experience	70.0%	85.6%	<0.001 ^a^
I received encouragement from a mentor to conduct the research for publications	71.3%	70.7%	0.282
My results with helpful for the scientific evidence	54.6%	78.7%	<0.001 ^a^

Note: Responses 3 and 4 in 4-point Likert scale were grouped as “agreement” for reporting purposes; ^a^ Mann-Whitney test was used to determine the significance, defined as *p* < 0.05.
